# Synthesis and biological evaluation of 3-arylcoumarins as potential anti-Alzheimer's disease agents

**DOI:** 10.1080/14756366.2019.1574297

**Published:** 2019-02-12

**Authors:** Jie Yang, Pingping Zhang, Yuheng Hu, Teng Liu, Jie Sun, Xiaojing Wang

**Affiliations:** a School of Medicine and Life Sciences, University of Jinan-Shandong Academy of Medical Sciences, Jinan, China;; b Institute of MateriaMedica, Shandong Academy of Medical Sciences, Jinan, China;; c Key Laboratory for Biotech-Drugs Ministry of Health, Jinan, China;; d Key Laboratory for Rare & Uncommon Diseases of Shandong Province, Jinan, China

**Keywords:** 3-Arylcoumarin, Alzheimer’s disease, cholinesterase inhibitors, monoamine oxidase inhibitors, antioxidant activity

## Abstract

Alzheimer's disease, a neurodegenerative illness, has the extremely complex pathogenesis. Accumulating evidence indicates there is a close relationship between several enzymes and Alzheimer's disease. Various substituted 3-arylcoumarin derivatives were synthesised, and their *in vitro* activity, including cholinesterase inhibitory activity, monoamine oxidase inhibitory activity, and antioxidant activity were investigated. Most of the compounds exhibited high activity; therefore 3-arylcoumarin compounds have the potential as drug candidates for the treatment of Alzheimer's disease.

## 1. Introduction

Alzheimer's disease (AD) has become one of the major diseases that threatening the health of elder people in modern society. Alzheimer's disease, a chronic progressive neurodegenerative disease, usually starts slowly and worsens over time. The most common early symptom is difficulty in remembering recent events. As the disease advances, symptoms can include problems with language, disorientation, loss of motivation, not managing and so on^1^. Gradually, bodily functions are lost, ultimately leading to death. AD is a refractory disease with complicated etiopathogenesis. Several hypotheses, such as cholinergic hypothesis, were proposed to explain the cause of the disease. The most currently available drug therapies were based on the cholinergic hypothesis, which proposes that AD is caused by reduced synthesis of the neurotransmitter acetylcholine (ACh)^2^.

The potential of acetylcholinesterase (AChE) inhibitors has been well explored and developed as an anti-AD drug^3^. There are currently five drugs used to treat AD cognitive problems, four of which are AChE inhibitors (tacrine, donepezil, galantamine, rivastigmine). The AChE activity of AD patients decreased, but butyrylcholinesterase (BuChE) activity was stable or even increased; the ratio of AChE to BuChE changed, indicating that BuChE may act as a compensatory mechanism for ACh metabolism. Studies have shown that selective BuChE inhibitors are beneficial for cognitive dysfunction of AD, and dual AChE/BuChE inhibition can maximise therapeutic efficacy^4^. Monoamine oxidase (MAO) is one of several enzymes that cause oxidative stress and dementia behaviour and psychological symptoms in AD. MAO-A inhibitors can be used as antidepressants and anxiolytics, while MAO-B inhibitors can be used alone or in combination to treat AD and Parkinson's disease^5^. Accumulating evidence shows that cholinesterase (ChE) and MAO are closely related to the disease symptoms and progression of AD. Many studies have identified the role of various ChE/MAO inhibitors in AD, while some showing positive results in clinical trials^6^.

3-Arylcoumarin refers to a class of compounds having a coumarin skeleton and having an aryl structure at the 3-position, which is rich in biological activity such as antioxidant^7^, anti-inflammatory^8^, antitumour^9^, antiviral^10^, antifungal^11^, antidiabetic^12^, MAO inhibitory activity^13^^,^^14^, ChE inhibitory activity^15^ and so on. The aetiology of Alzheimer's disease is complex, and for this multi-faceted disease, the efficacy of multi-targeted drugs is better than that of single-targeted drugs. Coumarin compounds, as dual inhibitors, are promising compounds that control AD^16–20^. Patil et al.^21^ reviewed the synthesis and designed aspects of coumarin derivatives as MAO inhibitors for AD, in which most 3-arylcoumarin compounds selectively inhibit MAO-B and give some guidance in modifying the structure. Wang et al.^15^ designed, synthesised, and evaluated a series of 6-substituted 3-arylcoumarin derivatives as dual AChE/MAO-B dual inhibitors for the treatment of AD, which provided meaningful information for further development of multifunctional drugs for AD treatment. Previous studies^15^^,^^21–24^ have designed coumarin derivatives as potential inhibitors of MAO and ChE, and have achieved good results. These results encourage us to further explore the potential of coumarins as candidates for the treatment of AD.

## 2. Experimental

### 2.1. Animals

The AB wild-type zebrafish was provided by Key Laboratory for Drug Screening Technology of Shandong Academy of Sciences. The zebrafish were cultured in an environment with a cycle of 14 h light and 10 h darkness, a pH of about 7.0, and a temperature of about 28 °C. The healthy zebrafish were mated in a tank one day before the experiment, with a male to female ratio of 1:1. The separator was taken the next day and the fertilised eggs were collected 0.5 h later. The fertilised eggs were washed 3 times with aquaculture water, then disinfected with methylene blue solution, and transferred to clean culture water of about 28 °C for light-control culture.

Wistar rat, weight 200–250 g, were obtained from Jinan Peng Yue Experimental Animal Co. (License number: SCXK (Lu) 2014–0007), Ltd. The animals were housed under standard laboratory conditions and maintained on a standard pellet diet and water *ad libitum*. All experiments involving living animals and their care were performed in strict accordance with the National Care and Use of Laboratory Animals by the National Animal Research Authority (China) and guidelines of Animal Care and Use issued by the University of Jinan Institutional Animal Care and Use Committee. The experiments were approved by the Institutional Animal Care and Use Committee of the School of Medicine and Life Sciences, University of Jinan. All efforts were made to minimise animal's suffering and to reduce the number of animals used.

### 2.2. *In vitro* cholinesterase inhibitory activity

The anticholinesterase activity of the 3-arylcoumarin compounds was determined by the method of Ellman et al.^25^ with slight modifications. AChE inhibitory activities were determined by AChE from electric eel (Macklin). BuChE inhibitory activities were determined by BuChE from horse serum (Aladdin). To a 10 ml tube, 2.65 ml of phosphate buffer solution (0.1 M, pH = 8.0), 100 µL of 5,5-dithiobis-2-nitrobenzoic acid (0.75 mM in 0.1 M phosphate buffer solution, pH = 8.0) were added sequentially, 50 µL AChE solution (0.2 U/mL in 0.1 M phosphate buffer solution, pH = 8.0), 100 µL of different concentrations of the sample solution, shaken well, and incubated at 37 °C for 5 min. Then 100 µL of substrate (1.5 mM in 0.1 M phosphate buffer solution, pH = 8.0) were added, shaken well, and incubated at 37 °C for 20 min. After the reaction was completed, 1 ml of sodium lauryl sulphate (4%, SDS in water) was added. The absorbance at 412 nm of the samples was measured using a spectrophotometer, and the inhibition rate of cholinesterase and the IC_50_ value of each sample were calculated according to the formula. Determination of the inhibitory activity of BuChE is similar. The sample blank group replaced the substrate with PBS, and the blank group replaced the sample solution with a solvent. The sample solution was set to five concentration gradients and the experiment was repeated three times. Donepezil was used as a positive control.

$$\text{Cholinesterase}\;\text{inhibitory}\;\text{effect}\;\left(\%\right)=\left[A_{0}-\left(A_{1}-A_{2}\right)\right]/A_{0}\times 100\%$$

where *A*_0_ is the absorbance of blank group; *A*_1_ is the absorbance of sample group; *A*_2_ is the absorbance of sample blank group.

### 2.3. *In vitro* monoamine oxidase inhibitory activity

The MAO inhibitory activity of the 3-arylcoumarin compounds was determined by the method of Holt et al.^26^ with slight modifications. The crude enzyme was extracted from the liver of 200–250 g of Wistar rats according to literature methods^26–28^. The crude enzyme protein content was determined by the Bradford/method using a Bradford Protein Assay Kit (Beyotime). Forty microlitres of enzyme solution and 40 µL of sample solution were added to a 96-well plate. The solution was then incubated at 37 °C for 20 min. One-twenty microlitres of the 4-(trifluoromethyl) benzylamine solution and 40 µL of the chromogenic agent (1 mmol/L vanillic acid, 0.5 mmol/L 4-aminoantipyrine, 4U/mL horseradish peroxidase, 0.2 M pH = 7.6 PBS constant volume) were added subsequently, and incubated at 37 °C for 90 min. The absorbance was measured at 490 nm using a microplate reader, and the inhibition rate of MAO and the IC_50_ value of each sample were calculated according to the formula. The control group replaced the sample solution with PBS (0.2 M, pH = 7.6), the positive control replaced the sample with the positive drug, and the blank group replaced the substrate with PBS, and each group was measured three times in parallel to average.

$$\text{Monoamineoxidase inhibitory effect}\;\left(\%\right)=\left[\left(A_{\text{C}}-A_{\text{B}}\right)-\left(A_{\text{S}}-A_{\text{SB}}\right)\right]/\left(A_{\text{C}}-A_{\text{B}}\right)\times 100\%$$

where *A*_C_ is the absorbance of control group; *A*_B_ is the absorbance of blank group; *A*_S_ is the absorbance of sample group; *A*_SB_ is the absorbance of sample blank group.

### 2.4. *In vitro* antioxidant activity

The total antioxidant capacity of the 3-arylcoumarin compounds was measured by the FRAP (the Ferric Reducing Ability of Plasma) assay of Benzie et al.^29^ with slight modifications. This method is based on the reduction of colourless Fe(III)-TPTZ(2,4,6-Tris(2-pyridyl)-s-triazine) complex to coloured Fe(II)-TPTZ complex by the compounds. FRAP working solution (300 mmol/L acetate buffer, 10 mmol/L TPTZ, 20 mmol/LFeCl_3_) was ready to use. A hundred microlitres of the sample solution was added to 3 ml of FRAP reagent and then incubated at 37 °C for 15 min. The experiments were repeated for three times. The absorbance was measured at 593 nm to clarify the changes. The standard curve was drawn with FeSO_4_ as standard material, and the regression equation was obtained. With 1.0 mmol/L FeSO_4_ as standard, the antioxidant activity of the sample is expressed in millimoles of Fe_2_SO_4_ required to achieve the same absorbance.

### 2.5. Zebrafish behavioural experiment

The experimental sample group and the blank control group were set in a 48-well plate, and 0.5 ml of aquaculture water and a juvenile fish that developed to 72 hpf (hours post fertilisation) were added to each well. Eight juvenile fish were set up for each experimental group. Compounds **2**, **20**, **22** were set at four different concentrations of 10 µg/ml, 50 µg/ml, 100 µg/ml, and 1000 µg/ml. The 48-well plate was placed in the dark box of the zebrafish behavioural analysis system. The fish were adapted to the environment for 10 min before the experiment. The zeblab software (Viewpoint, Lyon, France) was used to collect the trajectories of the juveniles in each group within 30 min, recorded every 5 min, and the total parade distance and parade time were exported by software. The average distance of each group of fish parades was calculated.

### 2.6. Statistical analysis

Data were shown as mean ± SD Differences between individual groups were analysed by using ANOVA followed by Dunnett's test. A difference with a *p* value of <.05 was considered to be significant.

## 3. Results and discussion

### 3.1. Chemistry

**Scheme 1.**
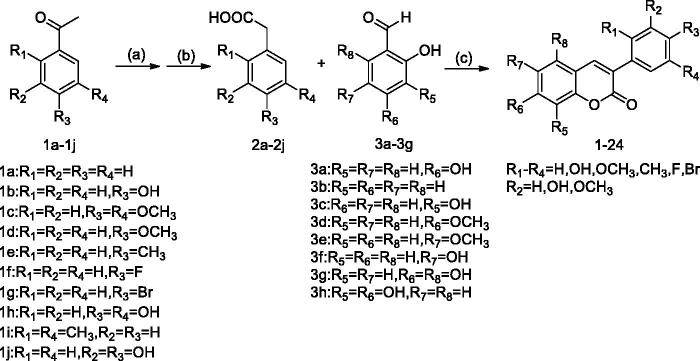
General synthetic route to 3-arylcoumarin, reagents, and conditions: (a) sulphur, p-toluenesulfonic acid, morpholine, 120 °C; (b) sodium hydroxide solution, tetrabutylammonium bromide, 100 °C; (c) acetic anhydride, triethylamine, 112 °C.

The synthetic methods and chemicals previously reported by our research group were used^30^. The synthetic route was summarised in Scheme 1. The substituted phenylacetic acid is synthesised by substituting acetophenone, then the 3-arylcoumarin structure skeleton is synthesised by Perkin reaction with substituted salicylaldehyde under the action of triethylamine and acetic anhydride, and the target compound is obtained by acid hydrolysis (Table 1). Details on the chemical and spectroscopic characterisations of compounds were described in the references.

**Table 1.** Compounds **1**–**24**.

Product	R_1_	R_2_	R_3_	R_4_	R_5_	R_6_	R_7_	R_8_
**1**	H	H	H	H	H	OH	H	H
**2**	H	H	H	H	OH	H	H	H
**3**	H	H	H	H	OH	OH	H	H
**4**	H	H	OH	H	H	OH	H	H
**5**	H	H	OH	H	H	H	H	H
**6**	H	H	OH	H	OH	H	H	H
**7**	H	H	OH	H	H	OH	H	OH
**8**	H	H	OH	H	OH	OH	H	H
**9**	H	H	OH	H	H	OCH_3_	H	H
**10**	H	H	OH	H	H	H	OCH_3_	H
**11**	H	H	OH	H	H	H	OH	H
**12**	H	H	OCH_3_	OCH_3_	OH	OH	H	H
**13**	H	H	OCH_3_	H	H	OH	H	H
**14**	H	H	OCH_3_	H	OH	H	H	H
**15**	H	H	OCH_3_	H	OH	OH	H	H
**16**	H	H	CH_3_	H	H	OH	H	H
**17**	H	H	CH_3_	H	OH	OH	H	H
**18**	H	H	F	H	H	OH	H	H
**19**	H	H	F	H	OH	OH	H	H
**20**	H	H	Br	H	H	H	H	H
**21**	H	H	Br	H	OH	OH	H	H
**22**	H	H	OH	OH	OH	OH	H	H
**23**	CH_3_	H	H	CH_3_	OH	OH	H	H
**24**	H	OH	OH	H	H	OH	H	H

### 3.2. Biological evaluation

#### 3.2.1. *In vitro* cholinesterase inhibitory activity

AChE is associated with neurons and axons, and is primarily responsible for ACh hydrolysis and termination. Studies have shown that long-term AChE inhibitor treatment can prevent the progression of cognitive dysfunction in patients with AD^31–33^. In the AD brain, BuChE may play a beneficial role by restoring cholinergic activity and/or by restoring AChE/BuChE activity ratio^34^. The AChE/BuChE inhibitory activity of all compounds was determined by the method of Ellman^25^, and donepezil was used as a reference compound in this assay. All the compounds were tested AChE/BuChE inhibitory activity *in vitro*, as shown in Table 2, most compounds presented inhibitory activity for AChE/BuChE. Most of the compounds exhibited moderate AChE inhibitory activity. Notably, compound **22** (IC_50_=3.04 ± 0.32 µM) had relatively strong activity, which displayed weaker capacity than donepezil, and compound **20** exhibited selective AChE inhibitory activity. Only compound **7** (IC_50_=2.76 ± 0.57 µM) had stronger BuChE activity than donepezil, and compounds **2**, **5**, **10** exhibited selective BuChE inhibitory activity. The results showed that 3-arylcoumarins with a R_5_, R_6_-dihydroxy group has a significantly stronger inhibitory activity than the R_6_- or R_5_-position mono-substituted 3-arylcoumarin, and 3-arylcoumarin having a R_5_, R_6_-dihydroxy group all showed high AChE inhibitory activity. However, there is no significant tendency for the inhibitory activity of BuChE.

**Table 2.** Biological evaluation *in vitro*.

Product	IC_50_ value (μM)			FRAP value(mmol/g)
	AChE inhibitory activity	BuChE inhibitory activity	MAO-B inhibitory activity	Antioxidant activity
**1**	78.49 ± 2.84	44.82 ± 2.35	>700	0.51 ± 0.09
**2**	>100	13.06 ± 0.03	>700	0.16 ± 0.05
**3**	17.97 ± 1.13	16.15 ± 2.18	73.45 ± 11.97	15.43 ± 0.19
**4**	105.42 ± 2.92	34.71 ± 1.87	>700	7.73 ± 0.12
**5**	>100	14.39 ± 1.94	>700	0.42 ± 0.01
**6**	68.25 ± 0.72	21.17 ± 0.65	>700	0.66 ± 0.08
**7**	16.39 ± 0.19	2.76 ± 0.57	204.61 ± 6.92	2.29 ± 0.02
**8**	13.13 ± 1.35	18.86 ± 0.43	65.75 ± 4.61	10.51 ± 0.23
**9**	100.59 ± 2.02	30.14 ± 3.06	>700	0.67 ± 0.04
**10**	>100	10.57 ± 0.85	>700	1.47 ± 0.04
**11**	40.37 ± 2.09	75.60 ± 4.01	>700	0.41 ± 0.08
**12**	8.45 ± 0.16	24.81 ± 3.81	63.36 ± 4.04	11.75 ± 0.12
**13**	42.44 ± 3.16	70.70 ± 5.75	>700	0.61 ± 0.11
**14**	84.57 ± 1.67	46.35 ± 6.78	503.05 ± 12.56	2.98 ± 0.22
**15**	13.31 ± 2.26	26.53 ± 0.98	562.06 ± 7.13	10.40 ± 0.48
**16**	82.81 ± 1.43	21.82 ± 2.54	>700	0.35 ± 0.05
**17**	12.58 ± 1.28	13.15 ± 0.35	>700	13.16 ± 0.68
**18**	74.29 ± 3.72	29.27 ± 4.41	>700	0.38 ± 0.07
**19**	27.12 ± 0.66	25.76 ± 3.61	231.62 ± 17.19	12.75 ± 0.65
**20**	19.21 ± 0.68	>100	>700	0.42 ± 0.06
**21**	8.75 ± 0.63	20.25 ± 1.60	>700	14.64 ± 0.53
**22**	3.04 ± 0.32	8.37 ± 0.68	27.03 ± 0.50	41.42 ± 0.35
**23**	13.74 ± 0.32	9.92 ± 0.54	>700	12.84 ± 0.13
**24**	30.90 ± 1.27	70.92 ± 4.22	330.04 ± 6.49	8.41 ± 0.11
Donepezil	0.021 ± 0.001	4.10 ± 0.18		
Rasagiline			0.125 ± 0.005	
Ascorbic Acid				3.79 ± 0.08

Each value represents the mean  ±  SD (*n* = 3).

#### 3.2.2. *In vitro* monoamine oxidase inhibitory activity

MAO plays an important role in neurotransmitter inactivation, and MAO dysfunction is considered to be the cause of many mental and neurological diseases. MAO plays an important role in AD pathology, and the progress of AD has a close correlation with MAO activity^35^^,^^36^. All the synthesised compounds were evaluated for their MAO inhibitory activity in the way of Holt^26^ by references. The selective MAO-B inhibitor rasagiline was used as a reference compound in this assay. The MAO used in the experiment was self-made. The protein content standard curve was drawn according to the absorbance of the protein standards at different concentrations at 595 nm, and then the crude enzyme protein content was obtained by a regression equation according to the absorbance of the crude enzyme. The crude enzyme obtained was 0.157 mg per mg of protein. Nearly half of the compounds showed MAO-B inhibitory activity. Among them, Compound **22** had the strongest inhibitory activity (IC_50_=27.03 ± 0.50 µM), which was weaker than the positive control drug rasagiline (IC_50_=0.125 ± 0.005 µM). The experimental results show that the 3-arylcoumarin compounds with R_5_, R_6_-dihydroxy group have better MAO inhibitory activity, indicating that R_5_, R_6_-dihydroxyl is very important for inhibiting MAO.

#### 3.2.3. *In vitro* antioxidant activity

Oxidative damage is involved in the pathogenesis of neuronal degeneration in AD, which may represent a potential therapeutic target for slowing the progression of AD or possibly preventing the onset of AD^37^. Oxidative stress is a key feature in determining AD. All the synthesised compounds were evaluated for their antioxidant activities by means of the Ferric Reducing Ability of Plasma. Vitamin C was used as a reference in this assay. The standard curve was drawn with FeSO_4_ as standard material, and the antioxidant capacity of the sample is expressed as FRAP value. As shown in Table 2, most of the compounds exhibited moderate to excellent antioxidant activities, even better than vitamin C. Especially, the FRAP values of compounds **3**, **8**, **12**, **15**, **17**, **19**, **21**, **22**, **23** were from 10.40 ± 0.48 to 41.42 ± 0.35 mmol/g, which were 3–11 folds of the value of vitamin C (FRAP value = 3.79 ± 0.08 mmol/g). Compound **22** (FRAP value = 41.42 ± 0.35 mmol/g) has the strongest antioxidant capacity. The antioxidant capacity of the compound is related to the position and number of hydroxyl substitution, and the R_5_, R_6_-dihydroxy substituted compound has a strong antioxidant capacity.

#### 3.2.4. Zebrafish behavioural experiment

**Figure 1.**
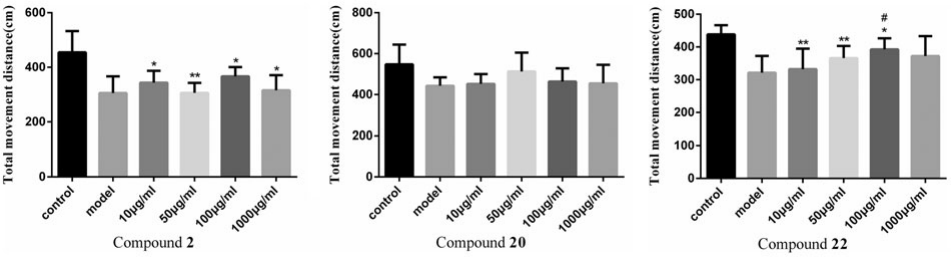
Effect of compounds **2**, **20**, **22** on the average total distance of zebrafish juveniles. (Compared with the control group, *p < .05 had a significant difference, **p < .01 had a very significant difference; compared with the model group, ^#^*p* < .05 had a significant difference, ^##^*p* < .01 had a very significant difference).

**Figure 2.**
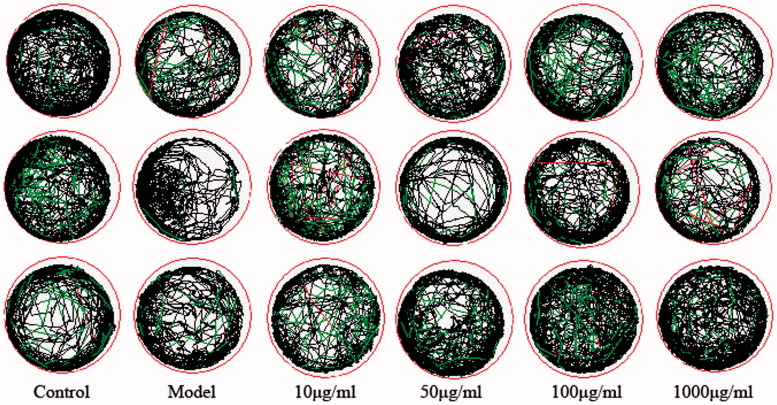
Statistical graph of the total trajectory of zebrafish juveniles exposed to different concentrations of compound **22** for 72 hpf.

To investigate the toxicity of compounds to zebrafish, the death and deformity of zebrafish were observed. Treatment in the blank group, 10, 50, 100, 1000 µg/ml group for three days did not cause malformation and death of zebrafish. Healthy zebrafish were AlCl_3_ inducted after 72 h of fertilisation to construct a model of zebrafish Alzheimer's disease. Compound **2** is a selective BuChE inhibitor and Compound **20** is a selective AChE inhibitor that did not show significant effects in zebrafish behavioural experiments. Compound **22** is not only a dual ChE inhibitor but also a MAO-B inhibitor, which exhibits certain effects in zebrafish behavioural experiments. In the range of 10–100 µg/ml, the total distance of zebrafish movement increases with the concentration of the compound increases. However, the high doses of compounds might cut down the movement distance of zebrafish (Figures 1 and 2).

## 4. Conclusions

We continue to study the pharmacological activities of 3-arylcoumarins synthesised by our research group. All the synthesised compounds were evaluated for their antioxidant activities in the way of the Ferric Reducing Ability of Plasma. Most compounds demonstrated moderate to high activity, among which compound **22** with several hydroxyl groups showed an excellent activity in this aspect. According to *in vitro* ChE inhibitory and MAO inhibitory test results, we selected selective AChE inhibitory compound **20**, selective BuChE inhibitory compound **2**, and dual ChE and MAO inhibitory compound **22** to study their anti-AD activity *in vivo*. Notably, compound **22** showed a certain effect *in vivo* experiments. Multi-target anti-AD compounds can modulate multiple signalling pathways or targets associated with AD, potentially producing significant clinical effects.

## Ethical statement

All experiments involving living animals and their care were performed in strict accordance with the National Care and Use of Laboratory Animals by the National Animal Research Authority (China) and guidelines of Animal Care and Use issued by the University of Jinan Institutional Animal Care and Use Committee. The experiments were approved by the Institutional Animal Care and Use Committee of the School of Medicine and Life Sciences, University of Jinan. All efforts were made to minimise animal’s suffering and to reduce the number of animals used.
